# Reply to Alvarez Stehle, E. Comment on “Leone et al. Association between Mediterranean Diet and Fatty Liver in Women with Overweight and Obesity. *Nutrients* 2022, *14*, 3771”

**DOI:** 10.3390/nu15051140

**Published:** 2023-02-24

**Authors:** Alessandro Leone, Simona Bertoli, Giorgio Bedogni, Laila Vignati, Marta Pellizzari, Alberto Battezzati

**Affiliations:** 1 International Center for the Assessment of Nutritional Status and the Development of Dietary Intervention Strategies (ICANS-DIS), Department of Food, Environmental and Nutritional Sciences (DeFENS), University of Milan, 20133 Milan, Italy; 2 Laboratory of Nutrition and Obesity Research, Department of Endocrine and Metabolic Diseases, Istituto Auxologico Italiano, IRCCS, 20145 Milan, Italy; 3 Department of Medical and Surgical Sciences, Alma Mater Studiorum-University of Bologna, 40126 Bologna, Italy; 4 Internal Medicine Unit Addressed to Frailty and Aging, Department of Primary Health Care, S. Maria delle Croci Hospital, AUSL Romagna, 48121 Ravenna, Italy; 5 Clinical Nutrition Unit, Department of Endocrine and Metabolic Diseases, Istituto Auxologico Italiano, IRCCS, 20145 Milan, Italy

We thank Dr. Elvira Alvarez Stehle [1] for her interest in our work reporting the association between a Mediterranean diet and a fatty liver in women with overweight and obesity [2]. 

Although deviations from the original protocol were found in the PREDIMED trial, they do not affect the validation of the MEDAS questionnaire. Schröder et al. [3] did, in fact, validate MEDAS against a 137-item semiquantitative FFQ using baseline data from PREDIMED prior to randomization. Certainly, the validation of MEDAS against an FFQ has several limitations. As Schröder et al. stated in their work [3], the similar designs of FFQ and MEDAS may have caused an overestimation of the accuracy of MEDAS given the likely between-method correlation of the errors.

More recently, García-Conesa et al. [4] performed a validation study of MEDAS using small samples of subjects from different Mediterranean and non-Mediterranean countries. They compared the results obtained from MEDAS with those obtained from a 3 day dietary record. In addition to confirming the reliability of the MEDAS questionnaire, they found a good agreement between the two methods, with values similar to, and in some cases greater than, those reported by Schröder et al. [3]. They found low agreement between the two methods for some food items, but this could be due to the difficulty of quantifying a weekly consumption using a 3 day dietary record. This limitation can be overcome by using multiple 7 day dietary records.

We thus believe that the MEDAS questionnaire remains a useful and reliable tool for rapidly assessing adherence to the Mediterranean diet. 
